# A vision for the future?

**DOI:** 10.1054/bjoc.2001.1984

**Published:** 2001-02-01

**Authors:** M Baum

**Affiliations:** Department of Psycho-oncology, University College London Medical School, 3rd Floor Bland Sutton Institute, 48 Riding House Street, London W1P 7PL UK

**Keywords:** adjuvant therapy, early breast cancer, anastrozole, ICI 182,780, goserelin, premenopausal, postmenopausal

## Abstract

Systemic adjuvant therapy is recommended immediately following surgical removal of the primary tumour in the majority of patients with early breast cancer, to prevent the recurrence of distant metastases. Significant progress has been made in the development and evaluation of endocrine therapies for systemic adjuvant therapy. In pre- and perimenopausal women, ovarian ablation has proven to be a valuable treatment option, though not always desirable for young patients. Thus, reversible medical ovarian suppression with a luteinizing hormone releasing hormone agonist, such as goserelin (Zoladex™), may provide an attractive alternative for such patients. International trials have indicated that goserelin provides an important addition to the choice of adjuvant therapies now available to pre- and perimenopausal patients. For postmenopausal patients, it is hoped that the ATAC (Arimidex, tamoxifen, alone or in combination) trial will reveal whether or not the benefits of anastrozole (Arimidex™) observed in advanced disease, where it has proven to be well tolerated and at least as effective as tamoxifen in recent trials, will translate to the early setting to provide further management options for these patients. On the horizon is yet another exciting endocrine agent, ICI 182,780 (Fulvestrant™), which has also been shown to be as effective as anastrozole in advanced disease. In terms of the future, these agents are likely to provide additional valuable treatment choices for early breast cancer across the patient spectrum. © 2001 Cancer Research Campaign


*British Journal of Cancer* (2001) **85**(Suppl 2), 15–18
© 2001 Cancer Research Campaign
doi: 10.1054/ bjoc.2001.1984, available online at http://www.idealibrary.com on IDEAL

### A vision for the future?

**M Baum**

Department of Psycho-oncology, University College London Medical School, 3rd Floor Bland Sutton Institute, 48 Riding House Street, London W1P 7PL UK

**Summary** Systemic adjuvant therapy is recommended immediately following surgical removal of the primary tumour in the majority of patients with early breast cancer, to prevent the recurrence of distant metastases. Significant progress has been made in the development and evaluation of endocrine therapies for systemic adjuvant therapy. In pre- and perimenopausal women, ovarian ablation has proven to be a valuable treatment option, though not always desirable for young patients. Thus, reversible medical ovarian suppression with a luteinizing hormone releasing hormone agonist, such as goserelin (Zoladex™), may provide an attractive alternative for such patients. International trials have indicated that goserelin provides an important addition to the choice of adjuvant therapies now available to pre- and perimenopausal patients. For postmenopausal patients, it is hoped that the ATAC (Arimidex, tamoxifen, alone or in combination) trial will reveal whether or not the benefits of anastrozole (Arimidex™) observed in advanced disease, where it has proven to be well tolerated and at least as effective as tamoxifen in recent trials, will translate to the early setting to provide further management options for these patients. On the horizon is yet another exciting endocrine agent, ICI 182,780 (Fulvestrant™), which has also been shown to be as effective as anastrozole in advanced disease. In terms of the future, these agents are likely to provide additional valuable treatment choices for early breast cancer across the patient spectrum. © 2001 Cancer Research Campaign

**Keywords**: adjuvant therapy; early breast cancer; anastrozole; ICI 182,780; goserelin; premenopausal; postmenopausal

### INTRODUCTION

Despite local treatment (surgery ± radiotherapy) for patients diagnosed with early breast cancer, approximately 30–40% of such patients will develop a distant recurrence of disease within 10 years of initial diagnosis (Early Breast Cancer Trialists' Collaborative Group, 1998; Rutqvist, 1998). Consequently, systemic adjuvant therapy is recommended for the majority of women alongside primary local treatment, with the aim of improving long-term survival. Over the past 10–15 years, there has been significant progress in the development of adjuvant therapies for patients with early breast cancer. Furthermore, there has been a dramatic fall of about 30% in breast cancer mortality rates in the UK between 1987 and 1997 (Peto, 2000). This is not attributed solely to adjuvant therapy but rather signifies an integrated approach to diagnosis, and the evaluation and adoption of new therapies.

### ENDOCRINE THERAPIES IN BREAST CANCER

In recent years, much has been elucidated in terms of the role of the endogenous hormones in the pathogenesis of breast cancer (Figure 1). Indeed, reflecting on the previous St. Gallen meeting, significant progress has been made in evaluating endocrine therapies for use in early breast cancer (Early Breast Cancer Trialists' Collaborative Group, 1998). At that time, tamoxifen (Nolvadex™) had undoubtedly been established as the standard for adjuvant endocrine therapy (immediately after local therapy) in hormone-sensitive pre- and postmenopausal patients with early breast cancer, and had also been suggested to be effective in the prevention of hormone-responsive breast cancers. Furthermore, luteinizing hormone releasing hormone (LHRH) agonists were recognized to be effective in advanced disease in pre- and perimenopausal women, and trials in early disease had been initiated. More specifically, a number of large trials involving the LHRH agonist, goserelin (Zoladex™), in early breast cancer had completed patient recruitment, with the first data awaited. Anastrozole (Arimidex™), an aromatase inhibitor, had been shown to be effective in advanced disease as second-line therapy for postmenopausal patients, first-line trials versus tamoxifen in the advanced disease setting had completed recruitment and the ATAC (Arimidex, tamoxifen, alone or in combination) trial to assess the efficacy and tolerability of Arimidex (anastrozole) in early disease had also been enrolling patients for 18 months. Additionally, ICI 182,780 (Fulvestrant™) had completed phase I and II studies, ultimately defining this agent as an oestrogen receptor (ER) down-regulator. This new endocrine agent was, at that time, a new candidate entering the metastatic disease setting.

Having now proven effective and well tolerated in advanced disease, in comparative or non-comparative trials, it is considered likely that each of these therapies could provide new treatment options across the spectrum of breast cancer disease. Emerging results from clinical trials indicate that these agents may be set to challenge clinical practice and to improve outcomes in the early disease setting that have traditionally been achieved using tamoxifen or chemotherapy.

### TAMOXIFEN – THE STANDARD ENDOCRINE THERAPY

Tamoxifen has long been established as an effective treatment option in postmenopausal patients with hormone-sensitive early breast cancer, providing a clear survival benefit when prescribed for 2–5 years following surgery (Early Breast Cancer Trialists' Collaborative Group, 1992; 1998), with 5 years of treatment providing the most benefit. In prevention of breast cancer, tamoxifen has been reported to provide a 49% reduction in the incidence of invasive breast cancer, in women at high risk from the disease,

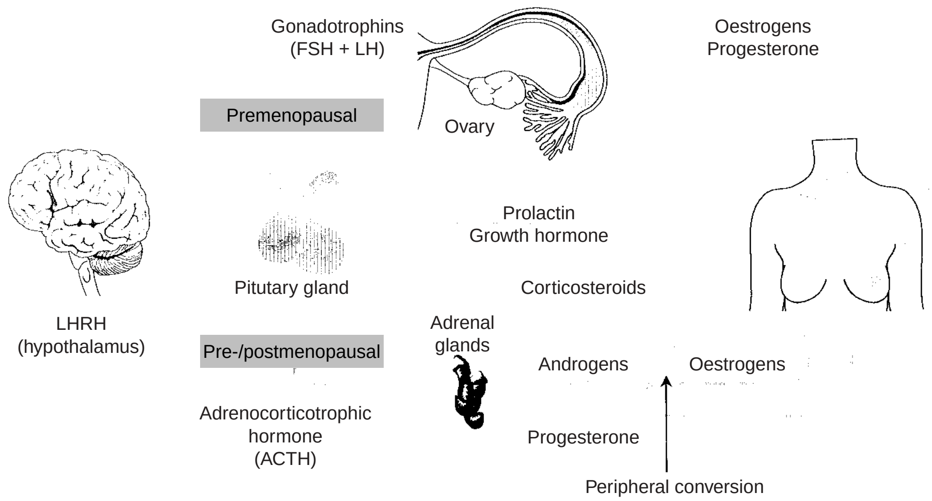

**Figure 1** Role of hormones and endocrine therapies in pre- and postmenopausal women with early breast cancer at 69 months' follow-up (Fisher et al, 2000). However, tamoxifen has been associated with treatment resistance, tumour flare and an increased incidence of thromboembolic events and long-term risk for endometrial cancer (Fisher et al, 1996). The effects of tamoxifen on the endometrium (postmenopausal bleeding and vaginal discharge) are an additional concern. These adverse effects are probably due to the partial agonist properties of tamoxifen, which may limit its potential utility as an antioestrogen (Katzenellenbogen et al, 1997). Moreover, although they are a concern, the risk of long-term adverse effects is more acceptable in patients receiving tamoxifen for management of the disease than when it is advocated for prevention of breast cancer.

### OPTIMIZING THERAPY FROM PRE- TO POSTMENOPAUSAL SETTINGS

The choice of adjuvant therapy for women with early breast cancer is heavily influenced by a number of prognostic and predictive factors (see Jonat, p 1), most notably the hormone-receptor status of the tumour – both ER and progesterone receptor (PgR).

Perhaps one of the key issues relating to endocrine therapies involves the treatment of ER-negative tumours. Current opinion suggests that there is no role for endocrine manipulation in ER-negative tumours. However, there are potentially three controversial roles for using endocrine agents in these patients:

- For secondary prevention – of contralateral breast cancer (Fisher et al, 1998)
- For protecting fertility in ER-negative women who are receiving chemotherapy – shutting down the ovary using LHRH agonists may protect future ovarian function (Boccardo et al, 1994; Teslik and Horejsi, 1997; Jonat, 1998)
- Oestradiol itself is genotoxic, thus the action of aromatase inhibitors (or possibly LHRH agonists) in reducing circulating levels of oestradiol may have an impact in terms of prevention on ER-positive and ER-negative cases. In contrast, tamoxifen has not yet proven to be effective in ER-negative tumours, though this could be a consequence of its unique mode of action associated with receptor binding (Fisher et al, 1998).

For treatment of hormone-sensitive primary breast cancer, depriving the tumour by way of blockade or suppression of oestrogen synthesis has proven valuable in prolonging survival (Early Breast Cancer Trialists' Collaborative Group, 1996). Successful achievement of oestrogen-suppression is influenced by the patient's menopausal status. In premenopausal women, the main source of oestrogen is provided by the ovaries. However, after the menopause, oestradiol is synthesized peripherally through aromatization of androgens (Masamura et al, 1994) (Figure 1). Thus, in manipulating endocrine involvement in breast cancer, we have to take these different mechanisms of oestrogen production into account and consider whether it is possible to optimize efficacy and tolerability by using the available therapies alone or in combination. In optimizing endocrine therapy for early breast cancer, we need to evaluate the newer agents in terms of their efficacy in advanced disease, their tolerability as judged against the standard and whether or not the dosing regimens are convenient.

### Pre-/perimenopausal setting

Goserelin provides reversible medical ovarian suppression by reducing serum oestradiol levels to postmenopausal values within 21 days of the start of therapy, thus offering premenopausal patients an attractive alternative to conventional irreversible ovarian ablation by oophorectomy (or irradiation). In pre-/perimenopausal women, adjuvant ovarian ablation plays an important role in enhancing disease-free and overall survival in patients with early breast cancer (Early Breast Cancer Trialists' Collaborative Group, 1996), with the induction of amenorrhoea serving as a significant prognostic factor in these patients. Recent data from ongoing international adjuvant trials indicate that goserelin may be used in a number of different clinical situations involving pre-/perimenopausal patients with early breast cancer.

The results of the ZEBRA ('Zoladex' Early Breast Cancer Research Association) trial have demonstrated that goserelin monotherapy is as effective as cyclophosphamide/methotrexate/5-fluorouracil (CMF) in pre-/perimenopausal patients with ER-positive, node-positive, early breast cancer (Kaufmann, 2001). Although chemotherapy is thought to induce its effect partly by ovarian suppression, it would be expected to have an additional cytotoxic effect and thus provide further benefits compared with goserelin therapy in premenopausal patients with ER-positive

© 2001 Cancer Research Campaign tumours. However, this does not appear to be true and there is no reasonable explanation for this observation. This suggests that goserelin is a viable alternative to chemotherapy following local therapy in these patients. Although cytotoxic chemotherapy may remain the first-choice treatment in some patients, its efficacy has been largely attributed to its effects on ovarian suppression, with achievement of amenorrhoea an indicator of this effect (Powles, 1998). However, achievement of amenorrhoea with chemotherapy can be highly variable (30–90%) and dependent on a number of factors, including the regimen used and the age of the patient (Bines et al, 1996).

Goserelin could offer benefits when used not only as monotherapy but alternatively as an adjunct to chemotherapy (ZIPP trial: 'Zoladex' in premenopausal patients). Irrespective of standard therapy (standard therapy = surgery and/or radiotherapy and/or chemotherapy and/or tamoxifen), the addition of goserelin in unselected patients produces a further benefit. Furthermore, when selecting groups, in those who are chemo-naive and those who are ER-positive the results are more profound (Houghton et al, 2000). The sub-group analyses for this study should be available by the end of the year. Other international trials (see Jonat, p 1) have shown that goserelin is also effective in improving recurrence-free survival when combined with tamoxifen after cyclophosphamide/adriamycin/5-fluorouracil (CAF) chemotherapy (Davidson et al, 1999), or when combined with tamoxifen as an alternative to CMF chemotherapy (Boccardo et al, 2000; Jakesz et al, 2001; Jonat et al, 2000).

### Postmenopausal setting

For the first time, large-scale trials with anastrozole have demonstrated the superiority of an endocrine agent over tamoxifen for treating postmenopausal patients with hormone-sensitive metastatic breast cancer (Buzdar et al, 2000; see Buzdar, p 6). Moreover, based on data from the advanced disease setting, and as predicted from its pharmacology, anastrozole has a more desirable tolerability profile than tamoxifen. Unlike the concerns that have previously been raised regarding the incidence of thromboembolism with tamoxifen – which is exacerbated when used after initial chemotherapy (Fisher et al, 1996; Pritchard et al, 1996) – anastrozole has been associated with significantly fewer thromboembolic events in trials in advanced disease. Furthermore, fewer patients experienced vaginal bleeding events with anastrozole compared with tamoxifen, a finding that could be indicative of anastrozole having less effect than tamoxifen on the endometrium (Nabholtz et al, 2000; Bonneterre et al, 2000). Demonstration of the superior efficacy and improved tolerability of anastrozole compared with tamoxifen in advanced disease gives the hope that these findings will translate to the early breast cancer setting. The ATAC trial has now completed recruitment, with over 9300 patients from 380 centres in 21 countries, and represents the largest adjuvant trial of its kind to date. A recent review by the Data Monitoring Committee recommended that the trial should continue and revealed that there was no excess of the predicted adverse events in any of the trial arms, thus the safety data to date look very promising. The predicted number of events that would allow the first formal efficacy analysis is expected to be reached during 2001.

Finally, ICI 182,780 is another major innovation in endocrine therapy on the horizon for postmenopausal women. ICI 182,780 lacks the partial agonist activity characteristic of tamoxifen (see Robertson, p 11). This potent anti-oestrogen is highly effective in down-regulating ER protein and ultimately blocking cell proliferation. The first data from ongoing trials of ICI 182,780 as a second-line therapy in advanced disease have been collated and they confirm that it is at least as effective and well tolerated as anastrozole and may offer a further potential therapeutic option for the adjuvant breast cancer setting in the future (Robertson et al, 2000).

### A VISION FOR THE FUTURE

Based on these new findings, it is important to consider the potential impact of these endocrine agents on treatment guidelines for breast cancer today, and in the very near future. There have been a number of treatment recommendations to date, most notably those outlined by the International Consensus Panel on the Treatment of Primary Breast Cancer (Goldhirsch et al, 1998). These guidelines were based on evidence from clinical trials available at that time, when the use of LHRH agonists in the treatment of early breast cancer was still under investigation.

As successful ovarian suppression is known to improve outcomes in premenopausal women, substantial change in the management of premenopausal women with ER-positive tumours is likely, particularly with the data emerging on LHRH agonists. The greatest challenge will be in establishing precisely how to use these agents. Although there are few comparative data available for goserelin in combination with chemotherapy, given the value of goserelin as monotherapy, and the increasing appreciation of the importance of achieving ovarian suppression, there may be a strong case for combining goserelin with chemotherapy on a routine basis.

At the last St. Gallen meeting the aromatase inhibitors were not even considered. While data for adjuvant therapy with anastrozole are not yet available, the data in advanced disease are very encouraging, proving it to be a potent, effective and well-tolerated agent which is at least as effective as tamoxifen, the current standard. If these benefits are shown by the ATAC trial to translate into the early disease setting, anastrozole is likely to be included in the consensus statement by the next St. Gallen meeting.

### CONCLUSION

The vision for the future is exciting and we are now in a position to review the recommendations right across the spectrum (Figure 2). For pre- and perimenopausal patients, it is crucial to recognize the importance of successful ovarian suppression. In light of the positive data reported from international trials, goserelin should be incorporated into the consensus to allow patients to be offered an informed choice of therapies.

Pre-/perimenopausal patients with ER-positive tumours may currently be offered treatment with: tamoxifen; tamoxifen and chemotherapy; or ovarian suppression (with or without tamoxifen). For the postmenopausal patient, tamoxifen remains the standard for ER-positive patients with a good prognosis, and chemotherapy combined with tamoxifen for patients with a poor prognosis. We also await with interest the potential of anastrozole, with or without tamoxifen, for the next St. Gallen consensus 3 years from now.

Although both chemotherapy and tamoxifen have undoubtedly had a significant impact on the outcome of patients with early breast cancer, there is still room for improvement in treatment modalities, and it is hoped that the emerging data for endocrine

© 2001 Cancer Research Campaign

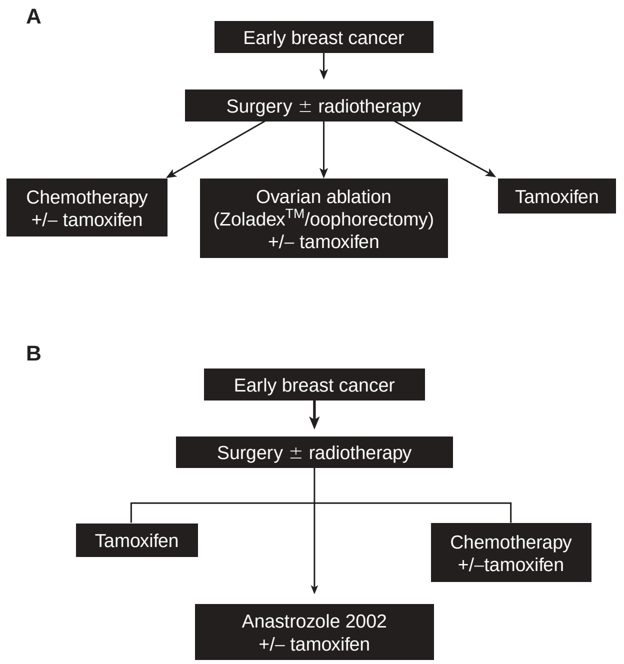

**Figure 2** Vision for the future of treatment across the patient spectrum: **(A)** pre-/perimenopausal patients **(B)** postmenopausal patients agents will provide more treatment options for both pre- and post-menopausal women. These newer, effective and well-tolerated endocrine agents – goserelin, anastrozole and ICI 182,780 – are likely to have an impact on and show improvements over tamoxifen treatment, which has been the standard treatment for breast cancer in postmenopausal women for the past 25 years.

### REFERENCES

Bines J, Oleske DM and Cobleigh MA (1996) Ovarian function in premenopausal women treated with adjuvant chemotherapy for breast cancer. *J Clin Oncol* **14**: 1718–1729

Boccardo F, Rubagotti A, Perrotta A, Amoroso D, Balestrero M, De Matteis A, Zola P, Sismondi P, Francini G, Petrioli R (1994) Ovarian ablation versus goserelin with or without tamoxifen in pre-perimenopausal patients with advanced breast cancer: Results of a multicentre Italian study. *Ann Oncol* **5**: 337–342

Boccardo F, Rubagotti A, Amoroso D, Mesiti M, Romeo D, Sismondi P, Giai M, Genta F, Pacini P, Distante V, Bolognesi A, Aldrighetti D and Farris A (2000) Cyclophosphamide, methotrexate, and fluorouracil versus tamoxifen plus ovarian suppression as adjuvant treatment of estrogen receptor-positive pre-/perimenopausal breast cancer patients: results of the Italian Breast Cancer Adjuvant Study Group 02 randomized trial. *J Clin Oncol* **18**: 2718–2727

Bonneterre J, Thurlimann B, Robertson JF, Krzakowski M, Mauriac L, Koralewski P, Vergote I, Webster A, Steinberg M and von Euler M (2000) Anastrozole versus tamoxifen as first-line therapy for advanced breast cancer in 668 postmenopausal women: Results of the Tamoxifen or Arimidex Randomized Group Efficacy and Tolerability Study. *J Clin Oncol* **18**: 3748–3757

Buzdar AU, Nabholtz JM, Robertson JFR, Thurlimann B, Bonneterre J, von Euler M, Steinberg M and Webster A (2000) Anastrozole (Arimidex™) versus tamoxifen as first-line therapy for advanced breast cancer (ABC) in postmenopausal women (Pm) – combined analysis from two identically designed multicentre trials. *Proc ASCO* **19**: 154 (Abst 609D)

Davidson N, O'Neill A, Vukov A, Osborne CK, Martino S, White D and Abeloff MD (1999) Effect of chemohormonal therapy in premenopausal, node-positive, receptor-positive breast cancer: an Eastern Cooperative Oncology Group Phase III Intergroup Trial (E5188, INT-0101). *Breast* **8**: 232–233 (Abst 69)

Early Breast Cancer Trialists' Collaborative Group (1992) Systemic treatment of early breast cancer by hormonal, cytotoxic or immune therapy. 133 randomized trials involving 31,000 recurrences and 24,000 deaths among 75,000 women. *Lancet* **339**: 1–15

Early Breast Cancer Trialists' Collaborative Group (1996) Ovarian ablation in early breast cancer: overview of the randomized trials. *Lancet* **348**: 1189–1196

Early Breast Cancer Trialists' Collaborative Group (1998) Tamoxifen for early breast cancer: an overview of the randomised trials. *Lancet* **351**: 1451–1467

Fisher B, Costantino J, Redmond C, Poisson R, Bowman D, Couture J, Dimitrov NV, Wolmark N, Wickerham DL and Fisher ER (1989) A randomized clinical trial evaluating tamoxifen in the treatment of patients with node-negative breast cancer who have estrogen-receptor-positive tumours. *N Engl J Med* **320**: 479–484

Fisher B, Dignam J, Bryant J and Wolmark N (1996) Five versus more than five years of tamoxifen therapy for breast cancer patients with negative lymph nodes and estrogen-receptor-positive tumors. *J Natl Cancer Inst* **88**: 1529–1542

Fisher B, Costantino JP, Wickerham DL, Redmond CK, Kavanah M, Cronin WM, Vogel V, Robidoux A, Dimitrov N, Atkins J, Daly M, Wieand S, Tan-Chiu E, Ford L and Wolmark N (1998) Tamoxifen for prevention of breast cancer: report of the National Surgical Adjuvant Breast and Bowel Project P-1 Study. *J Natl Cancer Inst* **90**(18): 1371–1388

Fisher B, Powles TJ and Pritchard KJ (2000) Tamoxifen for the prevention of breast cancer. *Eur J Cancer* **36**: 142–150

Goldhirsch A, Glick JH, Gelber RD and Senn HJ (1998) Meeting highlights: International Consensus Panel on the Treatment of Primary Breast Cancer. *J Natl Cancer Inst* **90**: 1601–1608

Houghton J, Baum M, Rutqvist LE, Nordensjkold B, Nicolucci A and Sawyer W (2000) The ZIPP trial of adjuvant Zoladex in premenopausal patients with early breast cancer: an update at five years. *Proc ASCO* **19**: 93a (Abst 359)

Jakesz R, Hausmaninger H, Samonigg E, Kubista E, Gnant C, Menzel T, Bauernhofer T, Seifert M, Haider K, Steger G, Mlineritsch B, Steindorfer P, Kwasny, Fridrik M and Wette V (2001) Complete endocrine blockade with tamoxifen and goserelin is superior to CMF in the adjuvant treatment of premenopausal, lymph node-positive and -negative patients with hormone-responsive breast cancer. *Breast* **10**(Suppl. 1): S10 (Abst 26)

Jonat W (1998) Luteinizing hormone-releasing hormone analogues – the rationale for adjuvant use in premenopausal women with early breast cancer. *Br J Cancer* **78** (Suppl 4): 5–8

Jonat W (2000) Zoladex™ versus CMF adjuvant therapy in pre-perimenopausal breast cancer: tolerability and amenorrhoea comparisons. *Proc ASCO* **19**: 87a (Abst 333)

Katzenellenbogen BS, Montano MM, Ekena K, Herman ME and McInerney EM (1997) William L. McGuire Memorial Lecture. Antiestrogens: mechanisms of action and resistance in breast cancer. *Breast Cancer Res Treat* **44**: 23–28

Kaufmann M (2001) Zoladex™ (goserelin) vs CMF as adjuvant therapy in pre-perimenopausal, node-positive early breast cancer: preliminary efficacy results from the ZEBRA study. *Breast* **10**(Suppl 1): S30 (Abst P53)

Masamura S, Adlercreutz H, Harvey H, Lipton A, Demers LM, Santen RJ and Santer SJ (1994) Aromatase inhibitor development for treatment of breast cancer. *Breast Cancer Res Treat* **33**: 19–26

Nabholtz JM, Buzdar A, Pollak M, Harwin W, Burton G, Mangalik A, Steinberg M, Webster A and von Euler M (2000) Anastrozole is superior to tamoxifen as first-line therapy for advanced breast cancer in postmenopausal women: Results of a North American Multicenter Randomized Trial. *J Clin Oncol* **18**: 3758–3767

Peto R (2000) UK and USA breast cancer deaths down 25% in year 2000 at ages 50–69. *Lancet* **317**: 1822

Powles TJ (1998) Prognostic impact of amenorrhoea after adjuvant chemotherapy. *Eur J Cancer* **34**: 603–605

Pritchard KI, Paterson AH, Paul NA, Zee B, Fine S and Pater J (1996) Increased thromboembolic complications with concurrent tamoxifen and chemotherapy in a randomized trial of adjuvant therapy for women with breast cancer. National Cancer Institute of Canada Clinical Trials Group Breast Cancer Site Group. *J Clin Oncol* **14**: 2731–2737

Robertson JFR, Nicholson RI, Anderson E, Dowsett M, Ellis I, Dixon JM, Bundred N, Webster A and Morris C (2000) The anti-tumor effects of single-dose, long-acting 'Faslodex' (ICI 182,780) compared with tamoxifen in post-menopausal primary breast cancer patients treated before surgery. *Breast Cancer Res Treat* **59**: 99

Rutqvist LE (1998) Controversial issues in adjuvant systemic therapy of early breast cancer. *Acta Oncol* **37**: 421–430

Teslik L and Horejsi J (1997) Ovarian protection in adolescent girls treated oncologically by inducing of prepubertal stage using LHRH analogs. *Acta Obstet Gynecol Scand* **76** (Suppl 167): 64 (Abst P56.17)

*© 2001 Cancer Research Campaign*
